# Biological impact of restrictive and liberal fluid strategies at low and high PEEP levels on lung and distal organs in experimental acute respiratory distress syndrome

**DOI:** 10.3389/fphys.2022.992401

**Published:** 2022-11-01

**Authors:** Nathane S. Felix, Ligia A. Maia, Nazareth N. Rocha, Gisele C. Rodrigues, Mayck Medeiros, Leticia A. da Silva, Camila M. Baldavira, Sandra de M. Fernezlian, Esmeralda M. Eher, Vera L. Capelozzi, Manu L. N. G. Malbrain, Paolo Pelosi, Patricia R. M. Rocco, Pedro L. Silva

**Affiliations:** ^1^ Laboratory of Pulmonary Investigation, Carlos Chagas Filho Institute of Biophysics, Federal University of Rio de Janeiro, Rio de Janeiro, Brazil; ^2^ Department of Physiology and Pharmacology, Biomedical Institute, Fluminense Federal University, Rio de Janeiro, Brazil; ^3^ Department of Pathology, School of Medicine, University of São Paulo, São Paulo, Brazil; ^4^ First Department of Anesthesiology and Intensive Therapy, Medical University of Lublin, Lublin, Poland; ^5^ Department of Surgical Sciences and Integrated Diagnostics (DISC), University of Genoa, Genoa, Italy; ^6^ San Martino Policlinico Hospital, IRCCS for Oncology and Neurosciences, Genoa, Italy

**Keywords:** acute respiratory distress syndrome, positive end-expiratory pressure, mechanical power, ventilator-induced lung injury, immunohistochemistry

## Abstract

**Background:** Fluid regimens in acute respiratory distress syndrome (ARDS) are conflicting. The amount of fluid and positive end-expiratory pressure (PEEP) level may interact leading to ventilator-induced lung injury (VILI). We therefore evaluated restrictive and liberal fluid strategies associated with low and high PEEP levels with regard to lung and kidney damage, as well as cardiorespiratory function in endotoxin-induced ARDS.

**Methods:** Thirty male Wistar rats received an intratracheal instillation of *Escherichia coli* lipopolysaccharide. After 24 h, the animals were anesthetized, protectively ventilated (V_T_ = 6 ml/kg), and randomized to restrictive (5 ml/kg/h) or liberal (40 ml/kg/h) fluid strategies (Ringer lactate). Both groups were then ventilated with PEEP = 3 cmH_2_O (PEEP3) and PEEP = 9 cmH_2_O (PEEP9) for 1 h (*n* = 6/group). Echocardiography, arterial blood gases, and lung mechanics were evaluated throughout the experiments. Histologic analyses were done on the lungs, and molecular biology was assessed in lungs and kidneys using six non-ventilated animals with no fluid therapy.

**Results:** In lungs, the liberal group showed increased transpulmonary plateau pressure compared with the restrictive group (liberal, 23.5 ± 2.9 cmH_2_O; restrictive, 18.8 ± 2.3 cmH_2_O, *p* = 0.046) under PEEP = 9 cmH_2_O. Gene expression associated with inflammation (interleukin [IL]-6) was higher in the liberal-PEEP9 group than the liberal-PEEP3 group (*p* = 0.006) and restrictive-PEEP9 (*p* = 0.012), Regardless of the fluid strategy, lung mechanical power and the heterogeneity index were higher, whereas birefringence for claudin-4 and zonula-ocludens-1 gene expression were lower in the PEEP9 groups. Perivascular edema was higher in liberal groups, regardless of PEEP levels. Markers related to damage to epithelial cells [club cell secreted protein (CC16)] and the extracellular matrix (syndecan) were higher in the liberal-PEEP9 group than the liberal-PEEP3 group (*p* = 0.010 and *p* = 0.024, respectively). In kidneys, the expression of IL-6 and neutrophil gelatinase-associated lipocalin was higher in PEEP9 groups, regardless of the fluid strategy. For the liberal strategy, PEEP = 9 cmH_2_O compared with PEEP = 3 cmH_2_O reduced the right ventricle systolic volume (37%) and inferior vena cava collapsibility index (45%).

**Conclusion:** The combination of a liberal fluid strategy and high PEEP led to more lung damage. The application of high PEEP, regardless of the fluid strategy, may also be deleterious to kidneys.

## Background

On admission to the intensive care unit (ICU), adequate administration of fluid and management of ventilatory parameters are important strategies applied to patients with acute respiratory distress syndrome (ARDS) (Griffiths et al., 2019). The reduction in mortality rate (36%) over a period of 17 years has been attributed to individualized fluid management during the first 7 days and protective mechanical ventilation, including the application of moderate to high positive end-expiratory pressure (PEEP) levels (Zhang et al., 2019). On admission to the ICU, a liberal fluid intake (∼30 ml/kg) is often used in septic shock, which may lead to a positive cumulative fluid balance (Peake et al., 2014; Yealy et al., 2014; Mouncey et al., 2015; Zhang et al., 2019) that may increase the risk of ventilator-induced lung injury (VILI) (Hotchkiss et al., 2000). However, a recent randomized clinical trial (RCT) showed no significant differences in 90-days mortality or serious adverse events among patients with septic shock who received restricted or standard fluid therapy in the ICU (Meyhoff et al., 2022). In addition to fluid management, PEEP can be set at moderate to high levels to promote alveolar recruitment/stabilization and improve functional residual capacity (Mauri, 2021), but this may increase static strain (Marini et al., 2020). As a result of this higher strain, ventilating stress may cross a threshold into a range that could damage highly jeopardized regions of the lung micro-architecture (Collino et al., 2019; Marini et al., 2020), leading to VILI. The biological effects of the combination of fluid strategy (restrictive or liberal) and low or high PEEP levels on lungs and kidneys require further elucidation. We hypothesized that, in the presence of a liberal fluid strategy, high PEEP would promote further lung damage in experimental ARDS. For this purpose, we evaluated the effects of liberal and restrictive fluid strategies associated with high and low PEEP levels on lung and kidney damage, as well as on cardiorespiratory function in a mild endotoxin induced ARDS model.

## Methods

### Study approval

This study was approved by the Animal Care and Use Committee of the Health Sciences Center, Federal University of Rio de Janeiro, Rio de Janeiro, Brazil (number 101/18). All animals received humane care in compliance with the *Principles of Laboratory Animal Care* formulated by the National Society for Medical Research and the U.S. National Academy of Sciences *Guide for the Care and Use of Laboratory Animals*. Conventional animals were housed at a controlled temperature (23°C) and in a controlled light-dark cycle (12–12 h), with free access to water and food. The study followed the *Animal Research: Reporting of In Vivo Experiments* (ARRIVE) guidelines for reporting of animal research (Percie du Sert et al., 2020).

## Animal preparation and experimental protocol

**FIGURE 1:**
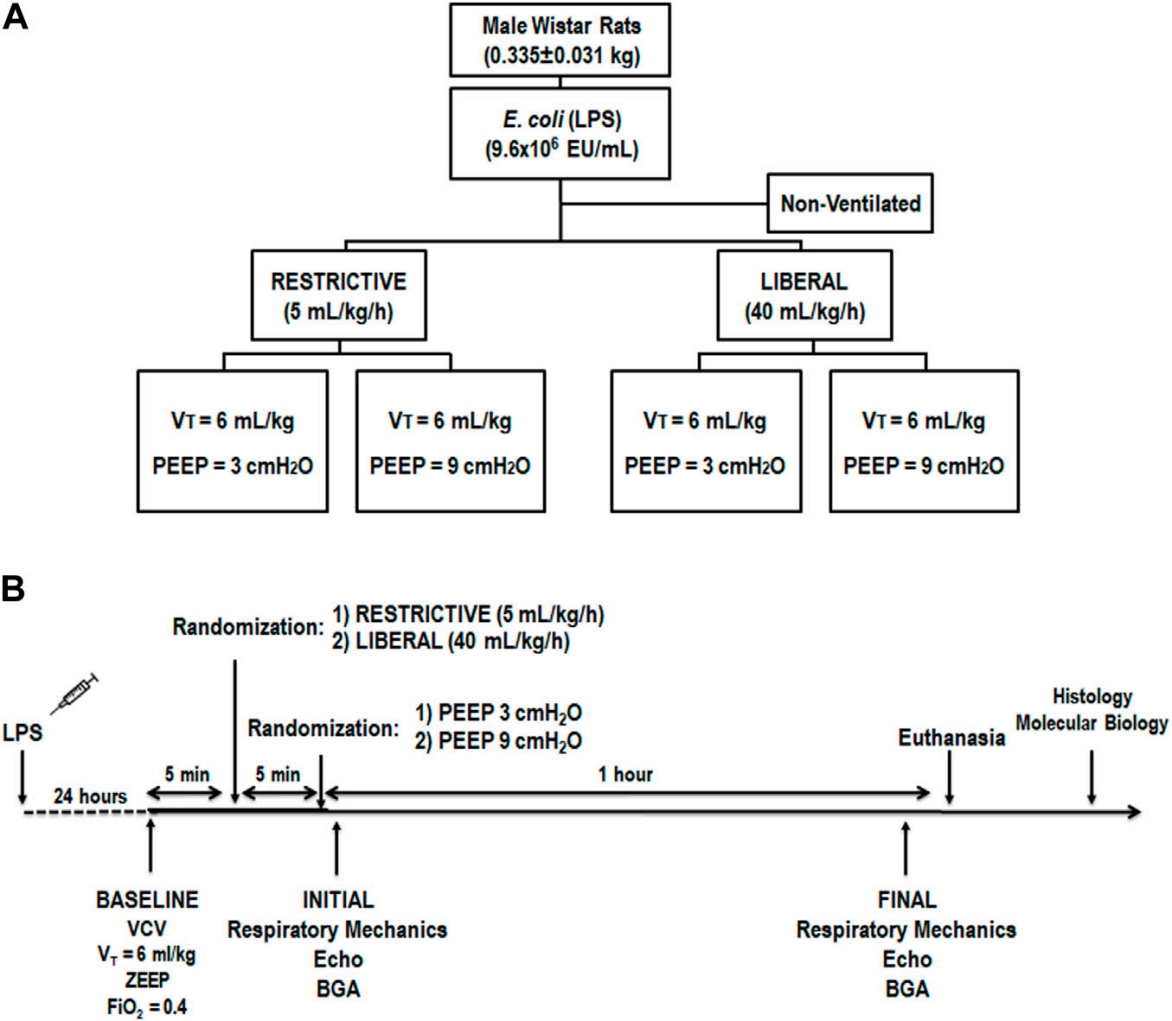
**(A)** Experimental design. The restrictive and liberal fluid strategies were set at 5 ml/kg/h and 40 ml/kg/h, respectively. *n* = 6 animals/group. **(B)** Temporal evolution. BGA, blood gas analysis; Echo, echocardiography; FiO_2_, inspired fraction of oxygen; LPS, *Escherichia coli* lipopolysaccharide; PEEP, positive end-expiratory pressure; VCV, volume-controlled ventilation; V_T_, tidal volume; ZEEP, zero end-expiratory pressure.

Beginning at 8:00 AM, 30 male Wistar rats (age 8–10 weeks, body weight 0.335 ± 0.031 kg) were used consecutively (Figure 1A). Rats were anesthetized by inhalation of 1.0% sevoflurane during spontaneous breathing (Sevorane; Cristália, Itapira, SP, Brazil) and received 9.6×10^6^ EU/mL *Escherichia coli* lipopolysaccharide (Merck Millipore, Burlington, MA, United States) diluted in 200 μL of saline solution intratracheally (i.t.) to induce experimental acute respiratory distress syndrome (ARDS) (Fernandes et al., 2022). After full recovery from anesthesia and a 24-h observation period, in the early morning (around 8:00 AM), animals were premedicated intraperitoneally (i.p.) with 10 mg/kg diazepam (Compaz; Cristália), followed by 100 mg/kg ketamine (Ketamin-S; Cristália) and 2 mg/kg midazolam (Dormicum; União Química, São Paulo, SP, Brazil). After administration of local anesthesia with 2% lidocaine (0.4 ml), a midline neck incision and tracheostomy were performed. An intravenous (i.v.) catheter (Jelco 24G; Becton, Dickinson, Franklin Lakes, NJ, United States) was inserted into the tail vein, and anesthesia was induced and maintained with midazolam (2 mg/kg/h) and ketamine (50 mg/kg/h). A second catheter (18G; Arrow International, Cleveland, OH, United States) was then placed in the right internal carotid artery for blood sampling and gas analysis (ABL80 FLEX; Radiometer, Copenhagen, Denmark) and to monitor the mean arterial pressure (MAP) (Networked Multiparameter Veterinary Monitor LifeWindow 6000V; Digicare Animal Health, Boynton Beach, FL, United States). A 30-cm-long water-filled catheter (PE-205; Becton, Dickinson) with side holes at the tip, connected to a differential pressure transducer (UT-PL-400; SCIREQ, Montreal, QC, Canada) was used to measure the esophageal pressure (Pes) (Saldiva et al., 1987; Coates et al., 1989). The catheter was passed into the stomach and then slowly withdrawn back into the esophagus; proper positioning was assessed using the occlusion test (Baydur et al., 1982). Heart rate (HR), MAP, and rectal temperature were monitored continuously. Body temperature was maintained at 37.5 ± 1°C using a heating bed. Animals in dorsal recumbency were paralyzed with pancuronium bromide (2 mg/kg, i.v.) and their lungs mechanically ventilated (Servo-I; MAQUET, Solna, Sweden) in volume-controlled mode (VCV) with constant inspiratory airflow, V_T_ = 6 ml/kg, respiratory rate (RR) to maintain V′_E_ = 160 ml/min, zero end-expiratory pressure (ZEEP), FiO_2_ = 1.0, and an inspiratory-expiratory ratio of 1:2 (baseline). Animals were randomized to receive a restrictive (∼5 ml/kg/h) or liberal (∼40 ml/kg/h) rate of fluid infusion of Ringer’s lactate (B. Braun, Crissier, Switzerland) *via* continuous intravenous infusion until the end of the study. The restrictive and liberal rates of fluid infusion of Ringer’s lactate were based on previous studies (Wiedemann et al., 2006; Holte et al., 2007; Rahbari et al., 2009; Evans et al., 2021). After the fluid strategy allocation, both groups were then exposed to PEEP three cmH_2_O (PEEP3) and PEEP nine cmH_2_O (PEEP9) for 1 h (*n* = 6/group). PEEP of three cmH_2_O was selected because it is consistent with functional residual capacity in rats during spontaneous breathing (Passaro et al., 2009), while PEEP of nine cmH_2_O was chosen based on a previous study that showed that this level promoted VILI but allowed survival for 2 h in this rodent model without severe hemodynamic impairment (Samary et al., 2015). After randomization, done using sealed envelopes, arterial blood gases, echocardiography, and lung mechanics were assessed at initial and final (after 1 h) time points. Heparin (1000 IU) was injected into the tail vein. All animals were euthanized by an overdose of sodium thiopental (60 mg/kg i.v.) and the trachea was then clamped at the final PEEP. Lungs and one kidney were then extracted for histology and molecular biology analysis. Six animals received lipopolysaccharide (LPS) intratracheally but were not mechanically ventilated or given fluids (non-ventilated [NV] animals) and after 24 h, they were euthanized and their lungs and one kidney removed for molecular biology analysis. The temporal evolution is shown in Figure 1B.

## Data acquisition and processing

Airflow, airway (Paw), and Pes pressures were recorded continuously throughout the experiments (Felix et al., 2019; Fernandes et al., 2022) using a computer running customer-made software written in LabVIEW (National Instruments; Austin, TX, United States). V_T_ was calculated as digital integration of the airflow signal. All signals were amplified in a four-channel signal conditioner (SC-24; SCIREQ), and sampled at 200 Hz with a 12-bit analog-to-digital converter (National Instruments). Peak transpulmonary pressure (Ppeak,_L_) was calculated as the difference between Paw and Pes; transpulmonary driving pressure (ΔP,_L_) was the difference between the transpulmonary plateau pressure at end inspiration (Pplat,_L_) and end expiration. Lung mechanical power (MP,_L_) was calculated according to the following formula (Gattinoni et al., 2016):

$${MP,}_{L}{=0.098\times RR\times V}_{T}{\times (Ppeak,}_{L}-(∆{P,}_{L}/2))$$

Mechanical data were computed offline by a routine written in MATLAB (version R2007a; MathWorks, Natick, MA, United States).

### Transthoracic echocardiography

Shaved animals were placed in the dorsal recumbent position. Transthoracic echocardiography was performed (Lang et al., 2015) by an expert (N.N.R.) blinded to the group allocation, using an UGEO HM70A system (Samsung, São Paulo, Brazil) equipped with a linear phased-array probe (8–13 MHz). Images were obtained from the subcostal and parasternal views. The following parameters were analyzed: right ventricular (RV) area, left ventricular (LV) area, and cardiac output (CO). Images of the inferior vena cava (IVC) were acquired in sagittal sections. To obtain a sagittal image, the probe was placed in the subxiphoid area and the liver was used as an acoustic window. Images of the IVC draining into the right atrium were obtained while the probe was placed in the subxiphoid area. The minimum diameter of the IVC on inspiration and the maximum diameter of the IVC on expiration were recorded using M-mode just beyond the point where the hepatic veins drain into the IVC. The IVC collapsibility (IVCC) index was calculated using the formula: IVCC index = maximum diameter on expiration—(minimum diameter on inspiration/maximum diameter on expiration) (Yildizdas and Aslan, 2020). All parameters followed the recommendations of the American Society of Echocardiography and the European Association of Cardiovascular Imaging (Lang et al., 2015).

### Lung histology

The lungs and heart were removed *en bloc*. The left lung was fixed in 4% formaldehyde and embedded in paraffin, cut longitudinally into three slices (each 4-μm thick) in the central zone using a microtome, stained with hematoxylin-eosin for histologic analysis (Felix et al., 2019), and assessed in a light microscope (Olympus BX51; Olympus Latin America, São Paulo, Brazil).

#### Quantification of heterogeneous airspace enlargement

Airspace enlargement was assessed by measuring the mean linear intercept between alveolar walls at ×400 magnification in the lung as a whole (Felix et al., 2019). External and internal border areas were not included in the analysis (Scaramuzzo et al., 2019) since the behavior of airspaces at different distances from the pleura cannot be observed clearly in rats. To characterize the heterogeneity of airspace enlargement, the central moment of the mean linear intercept (D_2_ of the mean linear intercept between alveolar walls) was computed from 20 noncoincident airspace measurements (Parameswaran et al., 2006), according to the following equation:

$$D_{2}=\mu \cdot \left(1+\frac{\sigma^{2}}{\mu^{2}+\sigma^{2}}\right)\cdot \left(2+\sigma \cdot \frac{\gamma }{\mu }\right)$$

where μ is the mean, σ is the variance of the airspace diameters, and γ is the skewness of the diameter distribution. After calculating D_2_, the heterogeneity index (β) was derived from the ratio of D_2_ and the mean linear intercept between the alveolar walls (Wierzchon et al., 2017). Quantification of heterogeneous airspace enlargement was performed by an investigator (L.A.S.) blinded to the group assignment.

##### Perivascular edema

To quantify perivascular edema, ten random, noncoincident microscopic fields containing venules were evaluated. The number of points falling on areas of perivascular edema and the number of intercepts between the lines of the integrating eyepiece and the basal membrane of the vessels were counted. The interstitial perivascular edema index was calculated as follows: number of points^1/2^/number of intercepts (Hizume et al., 2007). Quantification of perivascular edema was performed by an investigator (L.A.S.) blinded to the group assignment.

##### Claudin-4 quantification

To quantify the expression of claudin-4, the slides were digitally scanned at x40 magnification using a Pannoramic 250 whole slide scanner (3DHistech, Budapest, Hungary). The slides were analyzed using QuPath software (version 0.2.3; Centre for Cancer Research & Cell Biology, University of Edinburgh, Edinburgh, Scotland) using a semi-assisted method (Bankhead et al., 2017). QuPath outputted the number of positive cells per mm^2^ in all fields investigated. Quantification of claudin-4 was performed by an investigator (C.M.B.) blinded to the group assignment.

#### Molecular biology of lung and kidney

A quantitative real-time reverse transcription polymerase chain reaction (RT-PCR) was used. In lung tissue, gene expression of biomarkers associated with inflammation (interleukin [IL]-6), epithelial cell damage (club cell secretory protein 16 [CC16]), tight junction (zonula occludens [ZO]-1), and extracellular matrix (ECM) damage (syndecan) were measured. In kidney tissue, gene expressions of biomarkers associated with renal injury (neutrophil gelatinase-associated lipocalin [NGAL]) and inflammation (IL-6) were evaluated. The primer sequences are listed in Additional file 1 (Supplementary Table S1). Central slices of the right lung and kidney were cut, flash-frozen by immersion in liquid nitrogen, and stored at −80°C. For each sample, the expression of each gene was normalized to the acidic ribosomal phosphoprotein P0 (36B4) housekeeping gene (Akamine et al., 2007) and expressed as fold change relative to the NV group, using the 2^–∆∆Ct^ method, where ΔCt = Ct (target gene)—Ct (reference gene) (Schmittgen and Livak, 2008). Quantification of gene expression was performed by an investigator (M.M.) blinded to the group assignment.

## Statistical analysis

Sample size was calculated based on a previous study (Rocha et al., 2021) that detected differences in lung damage expressed as IL-6 gene expression. A sample size of six rats per group provided the appropriate power (1—β = 0.8) to identify significant differences in IL-6 expression, considering the effect size d = 2.0, a two-sided t test, and a sample size ratio of 1 (G*Power 3.1.9.2; University of Dusseldorf, Dusseldorf, Germany). The primary outcome was lung damage, expressed as IL-6 gene expression. The secondary outcomes were lung mechanics, airspace enlargement, perivascular edema, lung claudin-4 quantification, lung and kidney molecular biology, and echocardiography data.

Normality and equality of variance were evaluated by the Kolmogorov-Smirnov test with Lilliefors’ correction and Levene’s median test, respectively. Lung mechanics and arterial blood gas were compared by two-way ANOVA followed by Holm-Sidak’s multiple comparisons test. Histologic and molecular biology data were compared by the Kruskal-Wallis test followed by Dunn’s multiple comparisons test. Data that satisfied parametric assumptions were expressed as the mean and standard deviation and data that did not satisfy parametric assumptions as the median (interquartile range). Statistical significance was established at *p* < 0.05, two-tailed tests. All tests were carried out in GraphPad Prism 8.00 (GraphPad Software, La Jolla, CA, United States). Significance was established at *p* < 0.05.

## Results

No animal died during the experiments. The restrictive-PEEP3 and restrictive-PEEP9 groups reached a cumulative fluid infusion of 2.3 ± 0.7 ml and 2.4 ± 0.8 ml, respectively. The liberal-PEEP3 and liberal-PEEP9 groups received 13.1 ± 2.1 ml and 14.7 ± 5.2 ml, respectively (Table 1). At baseline, hemodynamic and oxygenation index did not differ among groups [Additional file 2 (Supplementary Table S2)]. At the end of the experiment (final), blood gas analysis did not differ between the groups (Table 1). RVSV and IVCC were lower in the liberal-PEEP9 group than the liberal-PEEP3 group (*p* = 0.007, and *p* = 0.001, respectively) (Table 2).

**TABLE 1:** Body weight, cumulative fluids, and mean arterial pressure at the final time point.

	Restrictive		Liberal	
	PEEP3	PEEP9	PEEP3	PEEP9
Body weight (kg)	0.332 ± 0.034	0.344 ± 0.038	0.309 ± 0.014	0.349 ± 0.020
Cumulative fluids (ml)	2.3 ± 0.7	2.4 ± 0.8	13.1 ± 2.1†	14.7 ± 5.2#
Fluid rate (ml/kg/h)	6.8 ± 1.4	7.0 ± 2.4	42.9 ± 6.9†	41.8 ± 13.3#
PaO_2_/FiO_2_	368 ± 154	467 ± 74	333 ± 120	461 ± 47
PaCO_2_ (mmHg)	34 ± 4	42 ± 8	33 ± 14	47 ± 14
HCO_3_ (mmol/L)	21 ± 2	21 ± 3	18 ± 6	19 ± 3
MAP (mmHg)	107 ± 25	126 ± 14	94 ± 24	108 ± 37

Comparisons were done by two-way ANOVA followed by Holm-Sidak’s multiple comparisons test (*n* = 6 animals/group). †Versus restrictive-PEEP3. #Versus restrictive-PEEP 9. *Restrictive* 5 ml/kg/h fluid strategy, *Liberal* 40 ml/kg/h fluid strategy, *PEEP* positive end-expiratory pressure, *PaO*_*2*_ */FiO*_*2*_ ratio of oxygen partial pressure in arterial blood and oxygen inspired fraction, *PaCO*_*2*_ arterial blood carbon dioxide partial pressure, *HCO*_*3*_ bicarbonate level, *MAP* mean arterial pressure.

**TABLE 2:** Echocardiography at the final time point.

Variables	Restrictive		Liberal	
	PEEP3	PEEP9	PEEP3	PEEP9
HR (bpm)	442 ± 73	420 ± 45	375 ± 56	398 ± 41
RVSV (ml)	0.272 ± 0.045	0.235 ± 0.057	0.360 ± 0.085	0.228 ± 0.036*
LVSV (ml)	0.215 ± 0.047	0.200 ± 0.041	0.234 ± 0.052	0.232 ± 0.029
CO (ml/min)	94 ± 17	95 ± 20	86 ± 12	92 ± 18
IVCC (%)	21.2 ± 7.3	18.3 ± 2.6	30.7 ± 4.7	17.1 ± 4.3*
RV area	0.275 ± 0.032	0.222 ± 0.059	0.338 ± 0.059	0.268 ± 0.069
LV area	0.165 ± 0.029	0167 ± 0.055	0.262 ± 0.092	0.228 ± 0.027

Comparisons were done by two-way ANOVA followed by Holm-Sidak’s multiple comparisons test (*n* = 6 animals/group). *Versus liberal-PEEP3. *Restrictive* 5 ml/kg/h fluid strategy, *Liberal* 40 ml/kg/h fluid strategy, *PEEP* positive end-expiratory pressure, *HR* heart rate, *RVSV* right ventricle systolic volume, *LVSV* left ventricle systolic volume, *CO* cardiac output, *IVCC* inferior vena cava collapsibility index, *RV* area right ventricle area, *LV* area left ventricle area.

At final, V_T_ and RR did not differ among the groups. In restrictive groups, Ppeak,_L_, Pplat,_L_ and MP,_L_ were higher with PEEP9 than PEEP3 (*p* < 0.001, *p* < 0.001, and *p* = 0.004; respectively). In liberal groups, Ppeak,_L_, Pplat,_L_, ΔP,_L_ and MP,_L_ were higher with PEEP9 than PEEP3 (*p* < 0.001, *p* < 0.001, *p* = 0.022, and *p* = 0.006; respectively). Pplat,_L_ was higher in the liberal group (23.5 ± 2.9 cmH_2_O) compared with the restrictive group (18.8 ± 2.3 cmH_2_O) with PEEP = 9 cmH_2_O (*p* = 0.049, Table 3).

**TABLE 3:** Lung mechanics at the final time point.

	Restrictive		Liberal	
	PEEP3	PEEP9	PEEP3	PEEP9
V_T_ (ml/kg)	6.4 ± 0.3	6.7 ± 0.9	6.5 ± 1.0	5.8 ± 1.0
RR (bpm)	73 ± 8	71 ± 6	76 ± 6	75 ± 4
Ppeak,_L_ (cmH_2_O)	15.1 ± 1.6	21.9 ± 2.3†	15.2 ± 1.5	25.7 ± 2.4*
Pplat,_L_ (cmH_2_O)	13.2 ± 1.8	18.8 ± 2.3†	13.5 ± 1.7	23.5 ± 2.9*#
ΔP,_L_ (cmH_2_O)	9.7 ± 1.5	9.6 ± 2.0	9.9 ± 1.5	13.6 ± 2.5*
MP,_L_ (mJ/min)	178 ± 35	286 ± 42†	184 ± 57	249 ± 40*

Comparisons were done by two-way ANOVA followed by Holm-Sidak’s multiple comparisons test (*p* < 0.05, *n* = 6 animals/group). *Versus liberal-PEEP3. †Versus restrictive-PEEP3. #Versus restrictive-PEEP 9 *Restrictive* 5 ml/kg/h fluid strategy, *Liberal* 40 ml/kg/h fluid strategy, *PEEP* positive end-expiratory pressure, *V*_*T*_ tidal volume, *RR* respiratory rate, *Ppeak,*_*L*_ transpulmonary peak pressure, *Pplat,*_*L*_ transpulmonary plateau pressure, *ΔP,*_*L*_ transpulmonary driving pressure, *MP,*_*L*_ lung mechanical power calculated according to the following formula MP,_L_ = 0.098 × V_T_ (L) × RR × [Ppeak,_L_ (ΔP,_L_/2)].

**FIGURE 2:**
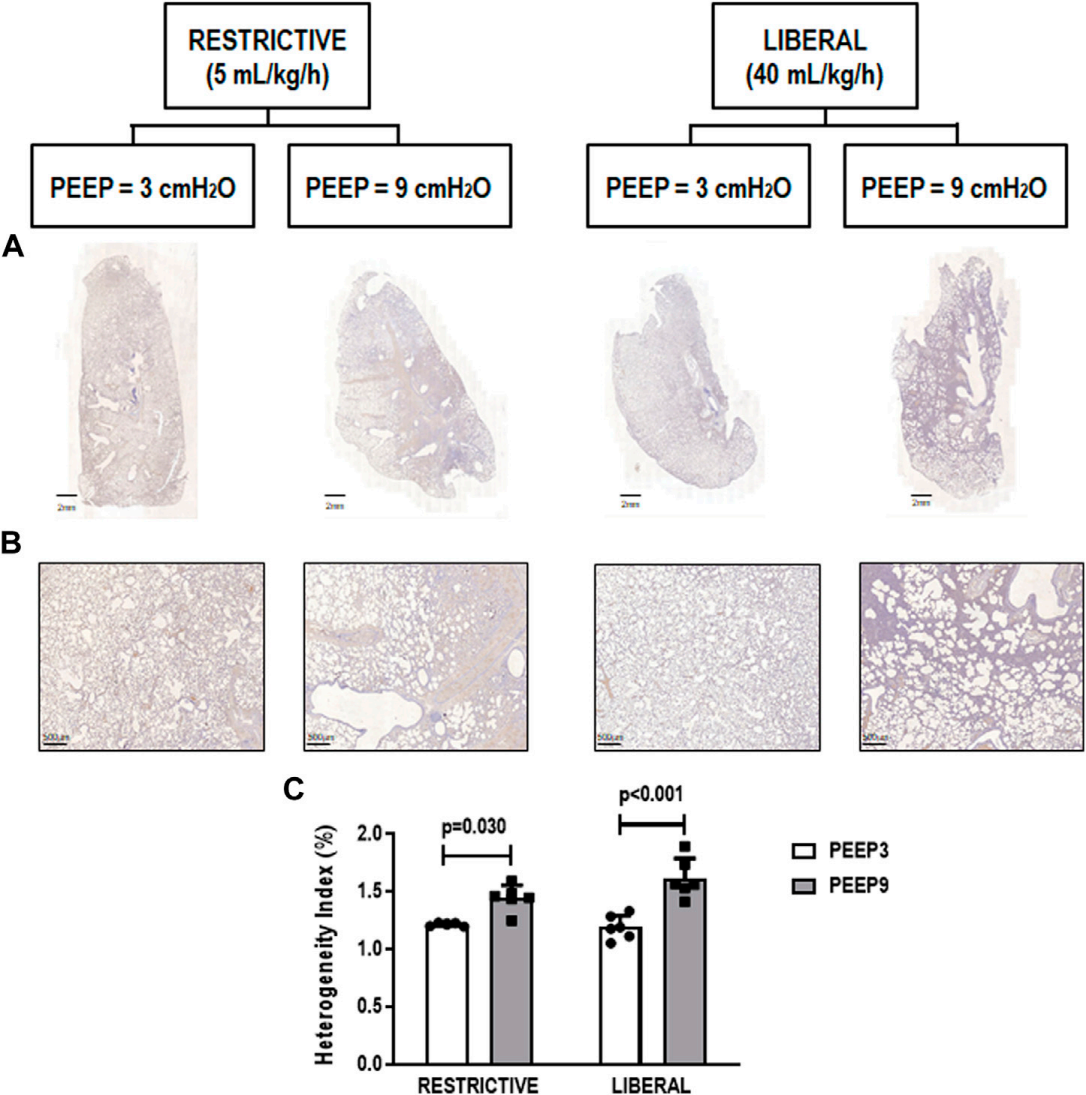
**(A)** Representative scanned whole lungs. **(B)** Histologic sections of alveolar pulmonary parenchyma (alveolar sacs and ducts, alveoli and associated capillary loops) stained by hematoxylin-eosin depicting quantification of the heterogeneity index in lung tissue. Scale bar, 500 μm. **(C)** Bars represent the mean and standard deviation of six animals/group. Comparisons were done with the Kruskal-Wallis test followed by Dunn’s multiple comparisons test (*p* < 0.05). The restrictive and liberal fluid strategies were set at 5 ml/kg/h and 40 ml/kg/h, respectively.

**FIGURE 3:**
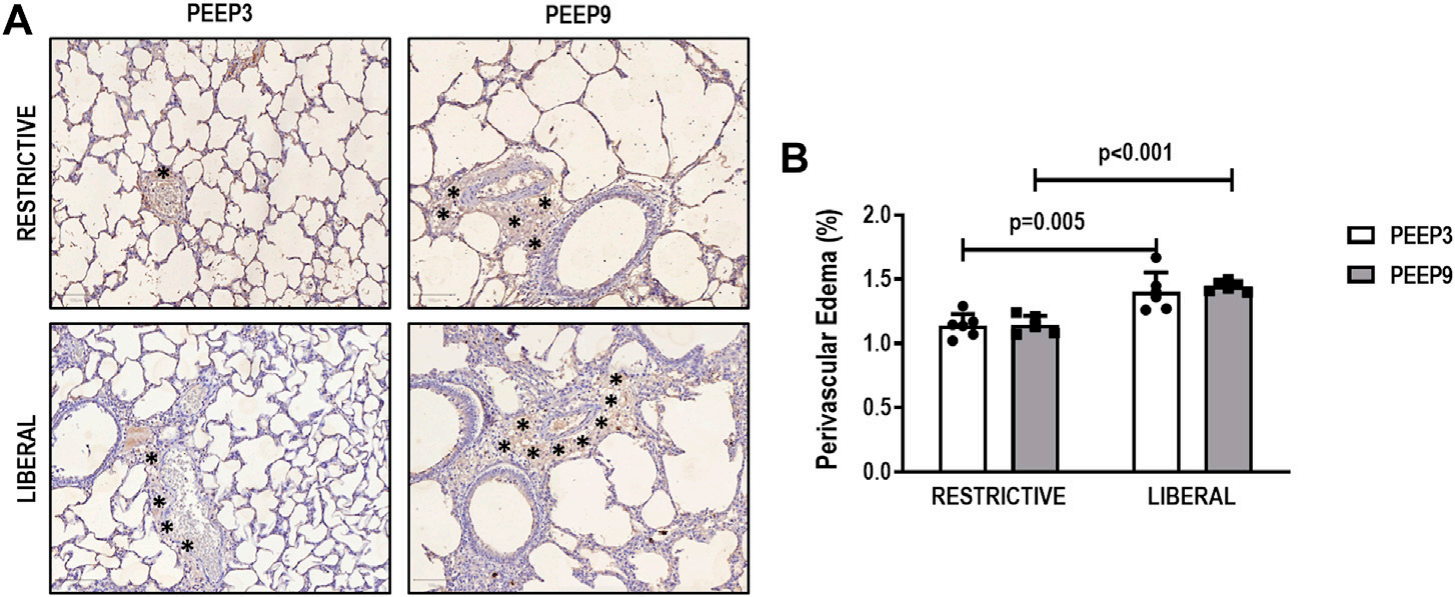
**(A)** Representative images of histologic sections of perivascular edema. Asterisks represent the perivascular edema. Scale bar, 100 μm. (**B)** Bars represent the mean and standard deviation of six animals/group. Comparisons were done with the Kruskal-Wallis test followed by Dunn’s multiple comparisons test (*p* < 0.05). The restrictive and liberal fluid strategies were set at 5 ml/kg/h and 40 ml/kg/h, respectively.

**FIGURE 4:**
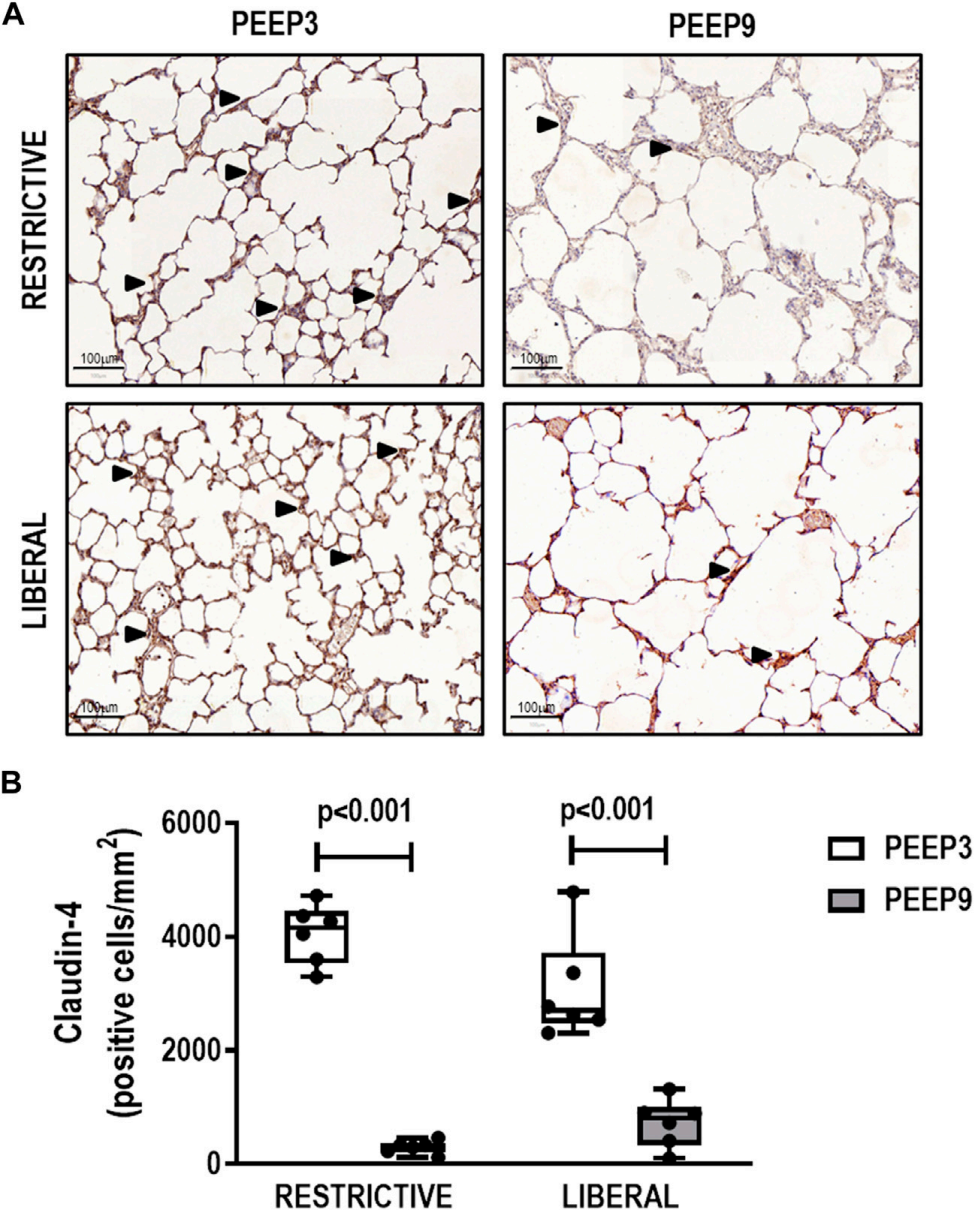
**(A)** Representative images of histologic sections of claudin-4 expression in lung tissue. Triangles represent claudin-4 expression in lung tissue (brown). Scale bar, 100 μm. (**B)** Boxplots represent the median and interquartile range of six animals per group. Comparisons were done with the Kruskal-Wallis test followed by Dunn’s multiple comparisons test (*p* < 0.05). The restrictive and liberal fluid strategies were set at 5 ml/kg/h and 40 ml/kg/h, respectively.

The heterogeneity index was higher in both PEEP9 groups compared with the PEEP3 groups, regardless of the fluid strategy (Restrictive: *p* = 0.030; Liberal: *p* < 0.001, respectively) (Figure 2). On the other hand, perivascular edema was higher in both liberal groups compared with the restrictive fluid groups, regardless of the PEEP levels (PEEP3: *p* = 0.005; PEEP9: *p* < 0.001, respectively) (Figure 3). Birefringence for claudin-4 in lung tissue was lower in both PEEP9 groups compared with PEEP3 groups, regardless of the fluid strategy (*p* < 0.001 for both) (Figure 4).

**FIGURE 5:**
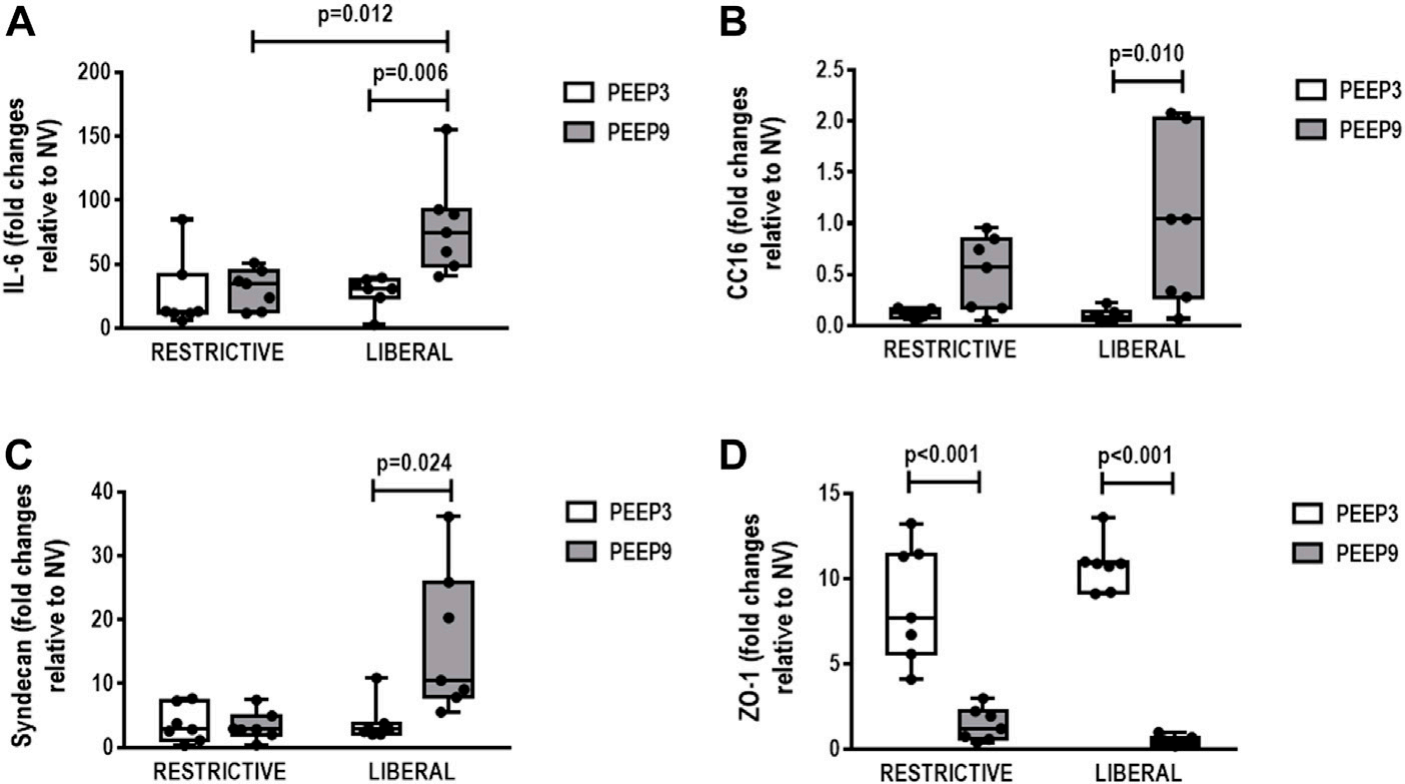
Gene expression of biomarkers associated with **(A)** inflammation (interleukin [IL]-6), **(B)** epithelial cell damage (club cell secretory protein 16, CC16), **(C)** extracellular matrix damage (syndecan), and **(D)** tight junction (zonula occludens [ZO]-1). Boxplots represent the median and interquartile range of six animals per group. Comparisons were done with the Kruskal-Wallis test followed by Dunn’s multiple comparisons test (*p* < 0.05). The restrictive and liberal fluid strategies were set at 5 ml/kg/h and 40 ml/kg/h, respectively.

In lung, IL-6 gene expression was higher in the liberal-PEEP9 group compared with the liberal-PEEP3 (*p* = 0.006) and restrictive-PEEP9 (*p* = 0.012) groups. Expression of CC-16 (*p* = 0.010) and syndecan (*p* = 0.024) genes was higher in the liberal-PEEP9 group than the liberal-PEEP3 group. ZO-1 gene expression was lower in both PEEP9 groups than PEEP3 groups, regardless of the fluid strategy (*p* < 0.001 for both) (Figure 5).

**FIGURE 6:**
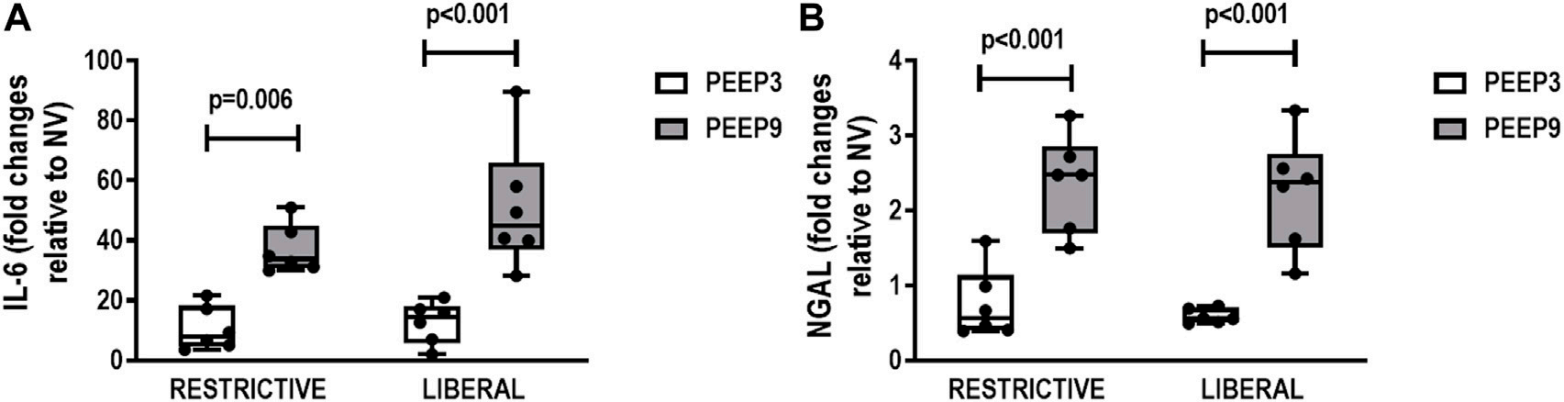
Gene expression of interleukin-6 (IL-6) **(A)** and neutrophilic gelatinase-associated lipocalin (NGAL) **(B)** in kidney tissue. Boxplots represent the median and interquartile range of six animals per group. Comparisons were done with the Kruskal-Wallis test followed by Dunn’s multiple comparisons test (*p* < 0.05). The restrictive and liberal fluid strategies were set at 5 ml/kg/h and 40 ml/kg/h, respectively.

In kidney, expression of IL-6 (Restrictive: *p* = 0.006; Liberal: *p* < 0.001, respectively) and NGAL (*p* < 0.001 for both) was higher in both PEEP9 groups, regardless of the fluid strategy (Figure 6).

## Discussion

### Overview

In the present mild experimental ARDS model, we found that: 1) PEEP9 increased lung mechanical power, and when combined with the liberal fluid strategy, Pplat,_L_ increased compared with the restrictive fluid strategy at equivalent PEEP9; 2) in lungs, inflammatory gene expression was higher in the liberal-PEEP9 group compared with the liberal-PEEP3 and restrictive-PEEP9 groups, whereas markers of epithelial cell and ECM damage were higher in the liberal-PEEP9 group than the liberal-PEEP3 group; 3) in both PEEP9 groups compared with PEEP3 groups, regardless of the fluid strategy, the heterogeneity index was higher, whereas birefringence for claudin-4 in lung tissue and ZO-1 gene expression were lower; 4) perivascular edema was higher in both liberal fluid strategy groups, regardless of the PEEP levels; 5) in kidneys, expression of IL-6 and NGAL genes was higher in both PEEP9 groups compared with the PEEP3 groups, regardless of the fluid strategy; 6) in the liberal fluid strategy groups, PEEP9 reduced right ventricle systolic volume and the IVCC index in comparison with PEEP3. Our data suggest that the combination of a liberal fluid strategy and high PEEP may lead to lung damage; application of high PEEP, regardless of the fluid strategy, may also be deleterious for the kidney. Animal models have been used to advance the field of VILI pathophysiology because direct and invasive measurements cannot be performed in humans. We used a model of *Escherichia coli* lipopolysaccharide induced-mild/moderate lung injury because it reproduces many changes in lung function and histology characteristic of human ARDS (Matute-Bello et al., 2011).

### Effects of PEEP in the lungs

The combination of increased alveolar pressure with increased capillary pressure may induce epithelial and endothelial cell damage at the level of the alveolar-capillary membrane (Kuebler et al., 1999). In our study, we observed that the application of a PEEP of nine cmH_2_O was able to increase lung mechanical power by changing the pressure domain, as observed in Ppeak, Pplat, and ultimately ΔP. We used a similar mechanical power formula used in a clinical setting. Mechanical power has been associated with VILI and increased mortality in both preclinical and clinical studies (Cressoni et al., 2016; Santos et al., 2018; Coppola et al., 2020). At high PEEP, static strain tends to increase, and due to repetitive ventilating stress, some alveolar units may cross a threshold into a range that could damage highly jeopardized regions of the lung’s micro-architecture (Marini et al., 2020; Mauri, 2021), leading to VILI. Recently, it was shown that increased static strain by changing the PEEP level may disrupt the intercellular junction, and ultimately alter lung impedance due to the increase in extravascular lung water during development of VILI (Steinberg et al., 2022). At similar high PEEP levels, the liberal fluid strategy was associated with higher Pplat compared with the restrictive fluid strategy. Pplat has been suggested as a surrogate parameter for positive cumulative fluid accumulation in critically ill patients due to an increase in the elastic properties of the abdominal cavity which represents one of the components of the chest wall, resulting in an increase in intrathoracic pressure and thus Pplat (Rosenberg et al., 2009). As the mechanical power was increased in the PEEP9 groups, the heterogeneity index, which measures heterogeneous alveolar enlargement, increased followed by a decrease in birefringence for claudin-4 in lung tissue, independently of the fluid infusion strategy. In others words, as alveoli disproportionally enlarge, the integrity of lung epithelial cells decreases. Birefringence for claudin-4 was measured in lung tissue because it is constitutively expressed in alveolar cells and is associated with preserved alveolar epithelial barrier function (Rokkam et al., 2011). Thus, the immunohistochemistry analysis was based on the amount of claudin-4 that was still preserved in lung tissue and not on claudin-4 synthesis, because our ventilatory protocol was short. The perivascular index was higher in those animals under the liberal fluid infusion strategy, and it represents only the juxtacapillary regions, closer to pre-acinar arterioles, which can be highly influenced by the fluid infusion strategy.

## Effects of the fluid strategy on the lungs

The restrictive and liberal fluid strategies were based on previous studies (Wiedemann et al., 2006; Holte et al., 2007; Rahbari et al., 2009; Evans et al., 2021). Although, there is a lack of consensus of liberal fluid strategy in clinical practice, the difference between the restrictive and liberal fluid strategies was close to that observed in surgical (Shin et al., 2018), septic shock (Meyhoff et al., 2022), and ARDS (Zhang et al., 2019) scenarios. A liberal fluid strategy in isolation was not enough to change the gene expression and histologic parameters. This may indicate that pulmonary vessels and capillaries are able to adapt through distension and capillary recruitment mechanisms. A recent RCT showed no significant differences in 90-days mortality or serious adverse events among patients with septic shock who received restricted or standard fluid therapy in the ICU (Meyhoff et al., 2022). However, lung damage increased only when a high fluid strategy was combined with increased alveolar pressure and high levels of mechanical power. This hypothesis was confirmed by increased gene expression of inflammatory, epithelial cell damage, and ECM markers. In addition, Zonula occludens decreased in both PEEP9 groups independently of the fluid strategy, likely due to impaired respiratory system and lung mechanics. Zonula occludens is encompassed within tight junction proteins. In addition to quantifying the remaining claudin-4 in lung tissue, ZO-1 gene expression was also measured as a way to observe whether there would be a gene signaling toward regeneration of epithelial cell integrity.

### Kidney injury markers

Expression of IL-6 and NGAL was higher with PEEP9 regardless of the fluid infusion strategy. In theory, PEEP during mechanical ventilation may impair renal function by altering renal hemodynamics or by increasing secretion of the antidiuretic hormone (Kaukinen and Eerola, 1979). Recently, a retrospective cohort study of patients with ARDS without kidney disease before the onset of ARDS, showed that peak airway pressure was associated with the severity of acute kidney injury (Panitchote et al., 2019). In the present study, we showed that peak airway pressure was higher in those animals ventilated under PEEP9 with both restrictive and liberal fluid strategies, and it may have influenced early biological markers related to kidney injury. Thus, the deleterious effects induced by PEEP were greater than those induced by an increased fluid strategy. Proactively administering a sufficient fluid volume is important during the early phases of a critical illness, as observed in previous clinical trials (Peake et al., 2014; Yealy et al., 2014; Mouncey et al., 2015). The recommendation of 30 ml/kg as an initial fluid volume (within the first 3 h of resuscitation) was set in the 2021 Surviving Sepsis Campaign Guidelines (SSCG) (Evans et al., 2021), according to the average volume of fluid before randomization in the ProCESS and ARISE trials. Furthermore, this was corroborated by a recent RCT on patients with septic shock in the ICU (Meyhoff et al., 2022).

### Hemodynamics

A mean fluid infusion rate as low as 6.8–7.0 ml/kg/h (restricitve-PEEP3 and restrictive-PEEP9, respectively) was sufficient to maintain MAP ≥70 mmHg and normal CO (Watson et al., 2004) values in the low groups, probably because intratracheal instillation of LPS, as opposed to sepsis-induced lung injury, does not produce severe hemodynamic instability (Famous et al., 2017). The difference between the liberal and restrictive strategies in terms of the fluid rate was close to those in previous studies (Holte et al., 2007; Shin et al., 2018). Although no major differences in CO and MAP were observed between the restrictive and liberal groups, the IVCC index was reduced in the liberal group mainly at PEEP9, which suggests fluid overload that could potentially increase the hydrostatic pressure in pulmonary capillaries. Previous studies have shown that PEEP levels applied to humans would be equivalent to two times higher than those applied in rats, according to the estimated transpulmonary pressure (Thammanomai et al., 2013). Therefore, the range of PEEP levels would be equivalent to 6 to 18 cmH_2_O, not uncommon in the ICU.

## Clinical implications

In critically ill patients with ARDS, lung protective and correct fluid strategies are cornerstones for improved clinical outcomes. As shown by Zhang et al. (2019), in a study of 5159 critically ill patients with ARDS enrolled in nine RCTs, the mortality rate decreased by 35.9% over a period of 17 years. They showed that at day 0, a substantial amount of fluids was infused in combination with application of moderate to high PEEP levels. During the following days (days 3–7), the fluid management strategy changed toward a more conservative strategy, in combination with lower tidal volumes, plateau pressures and moderate PEEP levels. Our preclinical study adds new pathophysiologic data in relation to the very early treatment in ARDS, when the application of high PEEP levels, likely due to high lung mechanical power, can change lung epithelial cell integrity very rapidly, independently of a liberal fluid strategy.

### Limitations

The fluid strategy rates used here were relatively high; nevertheless, similar fluid rates have been reported in clinical (Holte et al., 2007; Malbrain et al., 2018; Shin et al., 2018) and ARDS (Zhang et al., 2019) settings. Furthermore, in the present preclinical study, we aimed to evaluate groups with different fluid strategies to simulate the positive fluid balance observed at day 0 in patients with ARDS (Zhang et al., 2019). Although the duration of mechanical ventilation was relatively short, it should be normalized to the metabolism of small mammals whereby one rat hour approximately correspond to 27 human hours (Agoston, 2017). We did not compute the urine output throughout the experiment. Nevertheless, we may assume that there was likely a 10-fold increase in fluid overload in liberal compared to restrictive group. All animals were hemodynamically stable since this dose of LPS induced lung injury is not enough to cause significant hemodynamic instability (Fernandes et al., 2022). The level of IVCC was within 26%–27% that indicates hemodynamic stability, which is in agreement with our recently published study showing that IVCC ranged from 21 to 29% in rats after 24-h of intratracheal LPS (de Carvalho et al., 2022). Additionally, high PEEP is usually adjusted to counteract the lung edema due to fluid overload. Maybe extending the time during restrictive or liberal fluid strategy would better reflect the clinical setting. Nevertheless, the 1-h experiment allowed sufficient time to observe decreases in constitutive proteins at the alveolar-capillary membrane and changes in gene expression. It has been shown that there are no differences in susceptibility to VILI caused by sex (Lopez-Alonso et al., 2019). Rather than justify the use of animals of a single sex in experimental studies, these findings support the inclusion of both (Clayton and Collins, 2014). Thus, we could have used female animals and assumed a hypothesis of no influence of sex (Vutskits et al., 2019).

## Conclusion

The combination of a liberal fluid strategy and high PEEP levels led to increased lung damage, and the application of high PEEP may be deleterious for the kidneys, regardless of the fluid strategy.

## Data Availability

The original contributions presented in the study are included in the article/Supplementary Material, further inquiries can be directed to the corresponding author.
