# Supplementary material for: Biological impact of restrictive and liberal fluid strategies at low and high PEEP levels on lung and distal organs in experimental acute respiratory distress syndrome
Source: Front Physiol. 2022 Nov 1;13:992401. doi: 10.3389/fphys.2022.992401 (PMC9663484; doi:10.3389/fphys.2022.992401)
Supplement: Supplementary file 1 [file Table1.DOCX]

**Additional file 1: Table S1**

| Gene | Primer | Sequence (5′–3′) |
| --- | --- | --- |
| 36B4 | F | AAT CCT GAG CGA TGT GCA G |
|  | R | GCT GCC ATT GTC AAA CAC |
| IL-6 | F | CTC CGC AAG AGA CTT CCA G |
|  | R | CTC CTC TCC GGA CTT GTG A |
| CC-16 | F | GGC TTG CCG CTC CTT CTA TCA T |
|  | R | AGA TGC CTG GCA ATG TTG TGG A |
| Syndecan | F | GTT CCG CTG GTT TGT TGT TT |
|  | R | GAT GAA GGC TGT CCC AGG TA |
| NGAL | F | TTG GGA CAG GGA AGA ACG A |
|  | R | TCA ACG CTG GGC AAC ATT A |
| ZO1 | F | CAC CAC AGA CAT CCA ACC AG |
|  | R | CAC CAA CCA CTC TCC CTT GT |

Sequence of primers for the mediators evaluated and the housekeeping gene (36B4)

*36B4* acidic ribosomal phosphoprotein P0, *IL-6* interleukin 6, *CC-16* club cell protein 16, *NGAL* neutrophil gelatinase-associated lipocalin, *ZO-1* zonula occludens-1
