# Supplementary material for: Biological impact of restrictive and liberal fluid strategies at low and high PEEP levels on lung and distal organs in experimental acute respiratory distress syndrome
Source: Front Physiol. 2022 Nov 1;13:992401. doi: 10.3389/fphys.2022.992401 (PMC9663484; doi:10.3389/fphys.2022.992401)
Supplement: Supplementary file 2 [file Table2.DOCX]

Additional file 2 Table S2. Echocardiography and PaO_2_/FiO_2_ at the baseline

| Variables | Restrictive | | Liberal | |
| --- | --- | --- | --- | --- |
|  | PEEP3 | PEEP9 | PEEP3 | PEEP9 |
| HR (bpm) | 453 ± 38 | 426 ± 41 | 395 ± 30 | 423 ± 29 |
| RVSV (mL) | 0.322 ± 0.070 | 0.338 ± 0.034 | 0.282 ± 0.030 | 0.268 ± 0.037 |
| LVSV (mL) | 0.311 ± 0.062 | 0.299 ± 0.060 | 0.260 ± 0.030 | 0.267 ± 0.048 |
| CO (mL/min) | 140 ± 23 | 124 ± 33 | 113 ± 17 | 112 ± 15 |
| IVCC (%) | 27.3 ± 5.7 | 26.7 ± 6.9 | 26.9 ± 7.3 | 26.5 ± 3.2 |
| RV area | 0.328 ± 0.027 | 0.345 ± 0.023 | 0.286 ± 0.046 | 0.302 ± 0.027 |
| LV area | 0.230 ± 0.020 | 0.204 ± 0.038 | 0.194 ± 0.055 | 0.198 ± 0.027 |
| PaO_2_/FiO_2_ | 207 ± 66 | 202 ± 99 | 195 ± 107 | 197 ± 124 |

Comparisons were done by two-way ANOVA followed by Holm–Sidak's multiple comparisons test (n = 6 animals/group).

*Restrictive* 5 mL/kg/h fluid strategy, *Liberal* 40 mL/kg/h fluid strategy, *PEEP* positive end-expiratory pressure, *HR* heart rate, *RVSV* right ventricle systolic volume, *LVSV* left ventricle systolic volume, *CO* cardiac output, *IVCC* inferior vena cava collapsibility index, *RV* area right ventricle area, *LV* area left ventricle area, *PaO2/FiO2* ratio of oxygen partial pressure in arterial blood and oxygen inspired fraction
